# Elucidation of the recognition mechanisms for hemicellulose and pectin in *Clostridium cellulovorans* using intracellular quantitative proteome analysis

**DOI:** 10.1186/s13568-015-0115-6

**Published:** 2015-05-23

**Authors:** Shunsuke Aburaya, Kohei Esaka, Hironobu Morisaka, Kouichi Kuroda, Mitsuyoshi Ueda

**Affiliations:** Division of Applied Life Sciences, Graduate School of Agriculture, Kyoto University, Sakyo-ku, Kyoto, Japan; Kyoto Integrated Science and Technology Bio-Analysis Center, Shimogyo-ku, Kyoto Japan

**Keywords:** *Clostridium cellulovorans*, Proteome analysis, Monolithic column, Substrate recognition, Hemicellulose, Pectin, Metabolism

## Abstract

*Clostridium cellulovorans* is an anaerobic, cellulolytic bacterium, capable of effectively degrading and metabolizing various types of substrates, including cellulose, hemicellulose (xylan and galactomannan), and pectin. Among Clostridia, this ability to degrade and metabolize a wide range of hemicellulose and pectin substrates is a unique feature; however, the mechanisms are currently unknown. To clarify the mechanisms of hemicelluloses and pectin recognition and metabolism, we carried out a quantitative proteome analysis of *C. cellulovorans* cultured with these substrates. *C. cellulovorans* was cultured in the medium of glucose (control), xylan, galactomannan (Locus bean gum, LBG), or pectin for 36 h. Xylan and galactomannan were used to search for the common recognition mechanisms of hemicellulose, and pectin was used to search for unique recognition systems in *C. cellulovorans*. Using an isobaric tag method and liquid chromatograph/mass spectrometer equipped with a long monolithic silica capillary column, we identified 734 intracellular proteins from all substrates. We performed KEGG analyses and cluster analyses of the resulting proteins. In the KEGG analyses, we found common degradation mechanisms for hemicellulose and pectin. In the cluster analysis corresponding to the genome analysis, we detected substrate-specific clusters that include genes involved in substrate recognition, substrate degradation, and metabolism. Combining the results of the KEGG analyses and cluster analyses, we propose the mechanisms involved in the recognition and metabolism of hemicellulose and pectin in *C. cellulovorans*.

**Electronic supplementary material:**

The online version of this article (doi:10.1186/s13568-015-0115-6) contains supplementary material, which is available to authorized users.

## Introduction

There has been enormous recent interest in biorefinery using non-edible biomass as a source for renewable fuels (Lynd et al. 1999). To achieve this aim, it is necessary to convert various biological substrates into the desired chemicals and fuels (Saha 2003). Terrestrial biomass consists of cellulose, hemicellulose, and pectin (Cosgrove 2005). Cellulose is a polymer of *β*-glucose that constitutes the main backbone of the plant cell wall, and composes of about 50% of plant cell walls. Hemicellulose forms a primary network with cellulose microfibrils in various conformations, and the hemicellulose binds with pectin. Polysaccharides belonging to hemicellulose can be extracted from plant cell walls with alkali solution. Common hemicellulose polysaccharides include xyloglucan, xylan, and mannan. Pectin is a family of polysaccharides that include homogalacturonan, rhamnogalacturonan-I, and rhamnogalacturonan-II. Polysaccharides belonging to pectin can be extracted with hot water or chelating agents. Homogalacturonan is a major component of pectin. It is believed that pectin crosslinking involves calcium and boron ions (Cosgrove 2005).

Of these three components of the plant cell walls, the degradation of cellulose has been best studied. For example, *Clostridium thermocellum* can metabolize cellulose and is used for the degradation of cellulose to glucose (Prawitwong et al. 2013). *C. thermocellum* belongs to the Family 4 (Cluster III) of Clostridia (Rainey and Stackebrandt 1993; Collins et al. 1994), and genetically engineered strains of *C. cellulolyticum* can metabolize cellulose and xylan among hemicellulose, and are used for producing isobutanol from cellulose (Higashide et al. 2011). However, available strains cannot utilize all forms of hemicellulose and pectin. For maximally efficient biorefinery, utilization of hemicellulose and pectin is also essential.

*Clostridium cellulovorans* is a gram-positive, mesophilic, and cellulosome-producing anaerobe of the Family 1 (Cluster I) of Clostridia that can degrade and directly metabolize various kinds of carbohydrates such as cellulose, hemicellulose (xylan and mannan), and pectin (Petitdemange et al. 1984; Tamaru et al. 2010). Furthermore, *C. cellulovorans* alters its production of secreted enzymes, depending on which substrates are available (Morisaka et al. 2012; Matsui et al. 2013; Esaka et al. 2015). Thus, *C. cellulovorans* can distinguish among varied substrates and adapt to survive in different environments.

In Clostridia, the systems by which cells recognize different substrates have been widely studied. For example, *C. thermocellum* was reported to recognize the availability of cellulose through the coordinated action of sigma and anti-sigma factors with cellulose binding domains (Nataf et al. 2010). *C. cellulolyticum* was reported to recognize cellulose availability with a carbon catabolite repression mechanism and xylan availability with a two-component system (TCS) (Celik et al. 2013; Xu et al. 2013). These findings imply that substrate recognition systems can show great variety between species. However, the substrate recognition systems of *C. cellulovorans*, which can degrade and directly metabolize a greater variety of substrates than other Clostridia studied, are so far unknown. This suggests that *C. cellulovorans* may be an attractive model organism for the study of substrate recognition systems.

In the present study, we carried out an “intracellular” proteome analysis using an LC–MS/MS system equipped with a long monolithic silica capillary column (500 cm) (Aoki et al. 2013; Morisaka et al. 2012) in order to elucidate substrate recognition systems of *C. cellulovorans*. As substrates, we used xylan, galactomannan (locust bean gum; LBG), and pectin. Studies on the recognition and metabolism of galactomannan and pectin have not been performed in cellulosome-producing Clostridia because the commonly used model organisms, *C. cellulolyticum* and *C.thermocellum,* are unable to metabolize these substrates. Based on our findings, we propose metabolic systems for each substrate and molecular models of substrate recognition system in *C. cellulovorans*. We further suggest that hemicellulose and pectin were degraded outside of the cell with constitutively produced enzymes, such as cellulosomal proteins. We could clarify that the TCS response regulator AraC was used in the substrate recognition system common to both xylan and galactomannan. Interestingly, for pectin, *C. cellulovorans* regulated the hydrolase/isomerase pathway and the lyase/5-dehydro-4-deoxy-gluconate pathway via the Crp transcriptional regulator and the IclR family transcriptional regulator, respectively.

## Materials and methods

### Cell culture and media

*C. cellulovorans* 743B (ATCC35296) was grown anaerobically as previously described (Sleat et al. 1984), differing only in the carbon source, which was replaced by 0.3% (w/v) glucose (Nacalai Tesque, Kyoto, Japan), 0.3% (w/v) xylan from beechwood (Sigma, MO, USA), 0.3% (w/v) pectin from apple (Sigma), or 0.3% (w/v) LBG from *Caretonia silliqua* seeds (Sigma).

### Estimating the growth of the anaerobic bacteria

The growth curves of *C. cellulovorans* on each medium were determined by bacterial protein estimation, as previously described (Raman et al. 2009) with small modifications. Cells from 1 mL of cell culture were collected by centrifugation (13,000×*g*, 4°C, 10 min). The cell pellets were washed with 1 mL of phosphate-buffered saline (pH 7.4; PBS) and incubated with 800 μL of sodium deoxycholate (2%) for 20 min at 37°C. Two hundreds microliters of trichloroacetic acid (24%) was added to the suspension, which was then centrifuged (13,000×*g*, 4°C, 10 min). One hundred microliters of resolubilization solution (5% SDS, 2 N NaOH) was added to the suspension and vigorously mixed. The protein concentration was measured using a protein assay bicinchoninate kit (Nacalai Tesque), with bovine serum albumin used as a standard.

### Sample preparation for quantitative proteome analysis

*C. cellulovorans* was grown in 50 mL cultures to late-logarithmic phase (36 h). Cells were concentrated by centrifugation (6,000×*g*, 4°C, 10 min), and the supernatant was discarded. Cell pellets were collected by centrifugation (13,000×*g*, 4°C, 10 min) and washed with 500 µL PBS, and centrifuged again. Cells were resuspended with 500 µL of lysis buffer [2% (w/v) 3-(3-cholamidopropyl)dimethylammonio-1-propanesulfonate, 10 mM dithiothreitol, 1% (v/v) protease inhibitor cocktail for bacterial cell lysis (Sigma), 7 M urea, and 2 M thiourea in 50 mM Tris–HCl (Nacalai Tesque)]. The cells were disrupted with a Bioruptor UCD-250T (Cosmo Bio, Tokyo, Japan) at 250 W, 15 s on-and-off cycles for 10 min, on ice. The solution was centrifuged (13,000×*g*, 4°C, 20 min), and the supernatant was collected and subjected to ultrafiltration with an Amicon Ultra-0.5 Centrifugal Filter Unit (10 kDa, Millipore, MA, USA) and buffer-exchanged with 200 mM triethyl ammonium bicarbonate (TEAB; Sigma). Proteins were reduced by adding tris (2-carboxyethyl) phosphine to 10 mM from a 200 mM stock, and the reaction was allowed to proceed for 60 min at 55°C. Following the incubation, 5 µL of iodoacetamide (375 mM) was added, and the reaction continued for 30 min at room temperature in the dark. Proteins were precipitated by adding 1 mL of ice-cold acetone and incubating the solution overnight at −20°C. The precipitated proteins were resuspended with 100 µL of 200 mM TEAB, 2 µg of sequencing grade modified trypsin (Promega, WI, USA) was added, and incubated overnight at 37°C. The four samples for proteome analysis (glucose, xylan, pectin, and galactomannan) were labeled using a tandem mass tag (TMT) 6-plex labeling kit (Thermo Fisher Scientific, MA, USA) with reporters at *m*/*z* = 126, 127, 129, and 130, respectively, in 41 μL acetonitrile. After 60 min at room temperature, 8 μL of 5% (w/v) hydroxylamine was added to each tube and mixed for 15 min. As an internal standard for quantification, a mixture of tryptic fragments from all substrates was combined with TMT-131 (reporter at *m*/*z* = 131). Aliquots were pooled and evaporated under vacuum, then dissolved in 60 μL of formic acid (0.1%) and subjected to LC–MS/MS analysis.

### LC–MS/MS analysis

Proteome analysis was performed using an LC (Ultimate 3000; Thermo Fisher Scientific)–MS/MS (LTQ Orbitrap Velos Mass Spectrometer Thermo Fisher Scientific) system equipped with a long monolithic silica capillary column. Tryptic digests were separated by reverse-phase chromatography using a monolithic silica capillary column (500 cm long, 0.1 mm ID) (Morisaka et al. 2012), at a flow rate of 500 nL/min. A gradient was achieved by changing the ratio of the two eluents: eluent A, 0.1% (v/v) formic acid; eluent B, 80% acetonitrile containing 0.1% (v/v) formic acid. The gradient started with 5% B, increased to 45% B for 600 min, further increased to 95% B to wash the column for 140 min, returned to the initial condition, and was held for re-equilibration of the column. The separated analytes were detected using a mass spectrometer with a full scan range of 350–1,500 *m*/*z* (resolution 60,000), followed by 10 data-dependent higher-energy c-trap dissociation (HCD) MS/MS scans acquired for TMT reporter ions by using 40% normalized collision energy in HCD with 0.1 ms activation time in quantitative proteome analysis and with a full scan range of 350–1,500 *m*/*z* (resolution 60,000), followed by 10 data-dependent collision-induced dissociation (CID) MS/MS scans in a qualitative proteome analysis. An electrospray ionization (ESI) voltage was set at 2.3 kV. Triplicate analyses were performed for each sample in three independent experiments, and the collected data were reviewed for protein identification and quantification in a quantitative proteome analysis. Single analyses were performed for each sample of the three independent experiments in a qualitative proteome analysis. The collected data were reviewed for protein identification and quantification. Blank runs were inserted between runs of different samples.

### Data analysis

Data analysis was performed using Proteome Discoverer 1.4 (Thermo Fisher Scientific). Protein identification was performed using the Mascot algorithm against the *C. cellulovorans* protein database (4,254 sequences) from NCBI (National Center for Biotechnology Information, http://www.ncbi.nlm.nih.gov/), with a precursor mass tolerance of 20 ppm and a fragment ion mass tolerance of 50 mmu in quantitative proteome analysis, and mass tolerance of 2.0 Da and a fragment ion mass tolerance of 0.8 Da in qualitative proteome analysis. Carbamidomethylation of cysteine was set as a fixed modification for quantitative proteome analysis, and carbamidomethylation of cysteine and a TMT 6-plex at the N-terminus and lysine were set as fixed modifications for qualitative proteome analysis. Protein quantification was performed using the reporter ions quantifier with the TMT 6-plex method. The data were then filtered with a cut-off criteria of *q* value ≤0.05, corresponding to a 5% false discovery rate (FDR) on a spectral level. Proteins with no missing values in three replicates were accepted as identified proteins for the protein quantification analysis. In the qualitative proteome analysis, proteins with scores >10 were accepted as identified proteins. Global median normalization was performed to normalize the quantity of each tryptic digest injected into the mass spectrometer. The heat map was generated using Cluster 3.0 (de Hoon et al. 2004), which can carry out hierarchical cluster analysis (HCA). Euclidean distance was used to measure the similarities of the protein profile patterns within the clustering analysis. To visualize the clustering results from Cluster 3.0, Java TreeView software was used (Saldanha 2004). The pathway map was constructed from Kyoto Encyclopedia of Genes and Genomes (KEGG; http://www.genome.jp/kegg/) (Kanehisa and Goto 2000), and identified proteins were adapted to this map.

## Results

**Figure 1:**
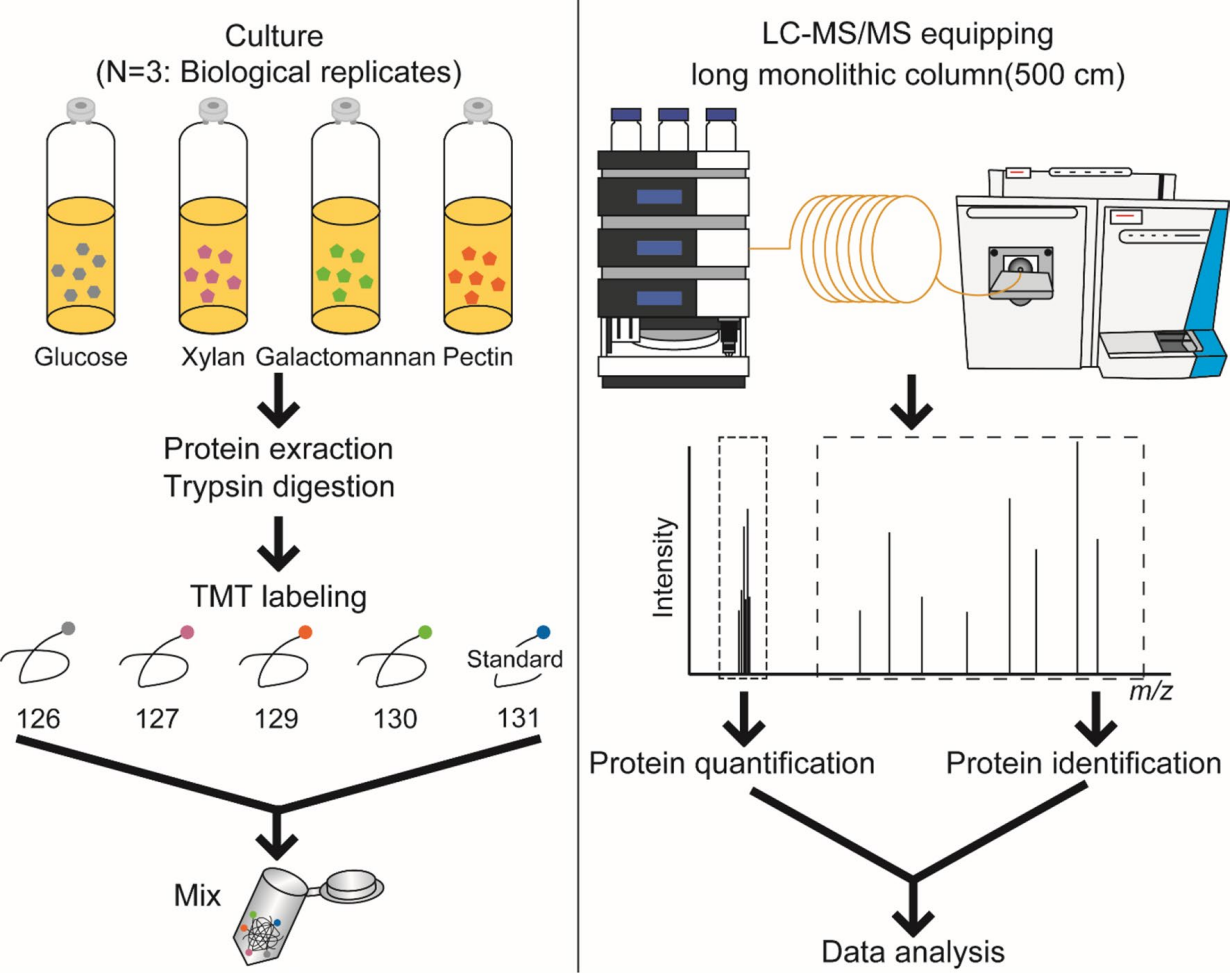
Experimental procedure of *C. cellulovorans* intracellular quantitative proteome analysis. Proteins prepared from cell lysates of *C. cellulovorans* grown in the presence of glucose, xylan, galactomannan (LBG), or pectin were individually reductive-alkylated and digested with trypsin. Tryptic fragments were labeled with tandem mass tags (TMTs). The labeled peptides were mixed and injected into the LC–MS/MS system with a long monolithic silica capillary column for mass measurement and collected data were used for protein quantification.

To identify substrate recognition systems of *C*. *cellulovorans*, we carried out a quantitative proteome analysis of cells grown anaerobically on media with the carbon sources-glucose, xylan, galactomannan (LBG), or pectin. Using a quantitative proteome approach based on isobaric tagging, we obtained protein profiles from cells grown on each carbon source. The workflow of the “intracellular” proteome analysis is illustrated in Figure 1.

### Growth confirmation

**Figure 2:**
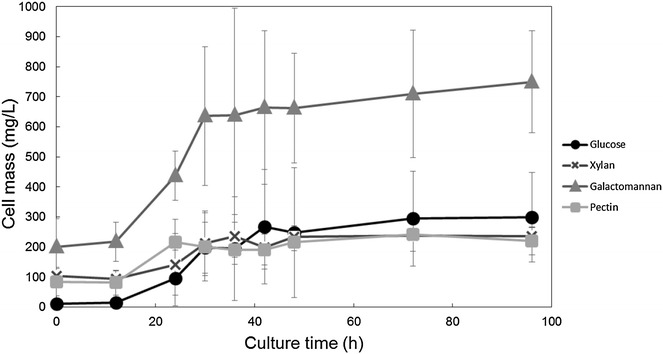
Confirmation of growth of *C. cellulovorans* cultured with four different substrates. Growth of *C. cellulovorans* was measured by estimation of protein in cell lysates to determine an appropriate culture time (Raman et al. 2009). At 36 h, *C. cellulovorans* appears to be in the late-logarithmic phase in all four substrates (glucose, xylan, galactomannan, pectin). These conditions were used for the proteome analysis. *Error bars* indicate SD (*n* = 3).

To determine the growth of *C. cellulovorans* on each of the substrates, we conducted bacterial protein estimation (Figure 2). As has previously been shown, *C. cellulovorans* can grow on xylan, pectin, or galactomannan as the sole carbon source (Sleat et al. 1984), although other cellulosome-producing Clostridia, such as *C. thermocellum* and *C.cellulolyticum*, cannot grow on pectin and galactomannan (Petitdemange et al. 1984; Prawitwong et al. 2013). Growth on glucose, xylan, and pectin was slower than that on galactomannan, but cells were collected from all cultures at similar growth phases. From the growth analysis, we selected a culture time of 36 h, as cells were in the late-logarithmic phase, which was appropriate for proteome analysis in terms of growth phase and protein concentration.

### Qualitative and quantitative proteome analysis

**Figure 3:**
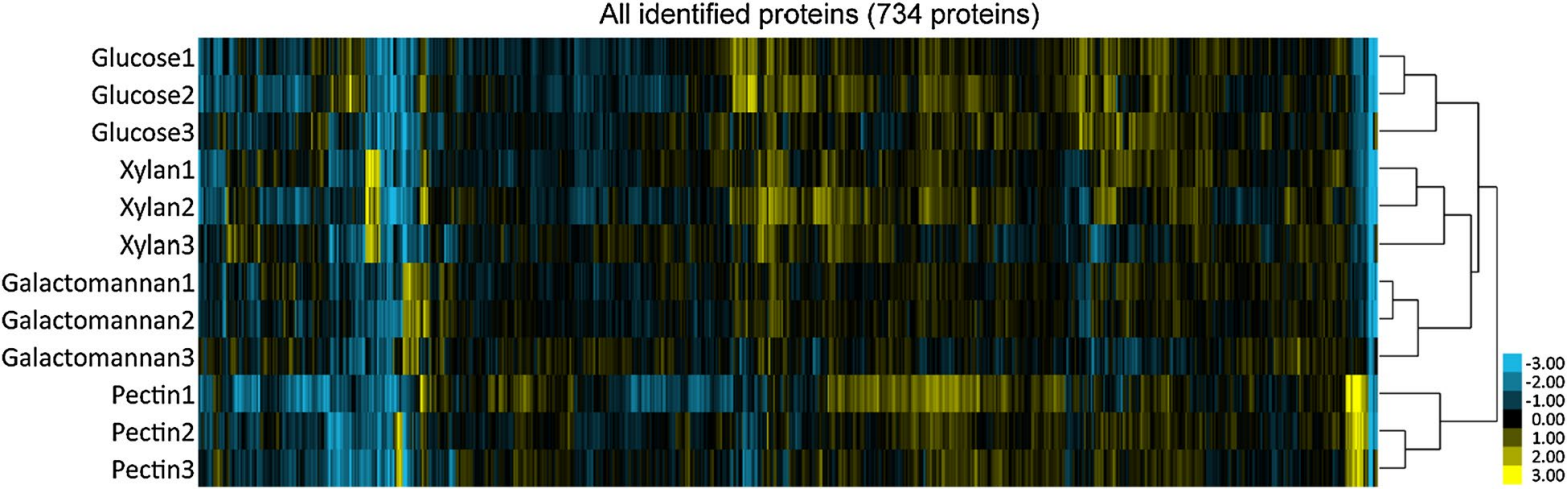
Hierarchical clustering analysis represented proteome profiles of *C. cellulovorans* with four different substrates. To standardize the data, the quantitative data were normalized using the median for each condition. The quantitative proteome data from three biological replicates of each substrate were used for the hierarchical clustering analysis. Each array was clustered in response to substrates, and each biological replicate was grouped together. This strategy ensures that the proteome analysis was biologically reproducible. *Color bar* indicates changes in protein abundance. Increased and decreased protein levels are shown in *yellow* and *blue*, respectively.

LC–MS/MS equipped with a long monolithic silica capillary using a tandem mass tag (TMT) 6-plex isobaric tag column was employed for intracellular quantitative proteome analysis. *C. cellulovorans* has 4,254 protein-encoding genes in its genome (Tamaru et al. 2010). For protein identification, we constructed a protein database built from the genome of *C. cellulovorans*. In total, we could identify 734 proteins from all samples within our cutoff criteria (Additional file 1: Table S1). To correct for variations in the amount of TMT-labeled peptides infused into the mass spectrometer, we normalized all quantitative data to the median value of that analysis. For all data analysis, we used these normalized relative quantification values. We checked for reproducibility of quantitative protein profiles among three biological replicates for each substrate by HCA (Figure 3). Each array was clustered individually based on each biological replicate. Our results indicate that each sample was reproducibly quantified, and analytical and biological replication of results was ensured. Furthermore, we separately carried out a qualitative proteome analysis to compare the results of quantitative proteome analysis (Additional file 2: Table S2).

### Substrate-specific proteins

To discover substrate-specific proteins (those that showed a significant change between different growth substrates), we carried out an empirical Bayes moderated *t* test (Smyth 2004). *P* values were adjusted with the Benjamini–Hochberg method to avoid the problem of multiple testing. The thresholds that we adopted were FDR-adjusted *p* value of <0.05 and fold-change of protein ratio >2.0 compared to glucose. Proteins for which the levels significantly changed were defined by comparison between glucose and each polysaccharide (xylan, galactomannan, and pectin) at these thresholds. All substrate-specific proteins detected are shown in Table 1. Using KEGG analysis and cluster analysis based on genome analysis, we focused mainly on metabolism-related and substrate recognition-related proteins for further analysis.

**Table 1:** Substrate-specific proteins

Specificity	Locus	Description in NCBI	Description in KEGG	Vs. Glucose	
				Log_2_ (fold change)	FDR-adjusted *p* value
Xylan	Clocel_0589	Alpha-l-fucosidase	Alpha-l-fucosidase 2	2.67	2.07E−04
	Clocel_0590	Xylose isomerase	Xylose isomerase	3.35	1.43E−04
	Clocel_0591	Transaldolase	Transaldolase	3.52	1.46E−06
	Clocel_0592	Xylulokinase	Xylulokinase	2.85	7.29E−05
	Clocel_1085	Dinitrogenase iron–molybdenum cofactor biosynthesis protein	–	2.32	4.24E−03
	Clocel_1151	Methyl-accepting chemotaxis sensory transducer	Methyl-accepting chemotaxis protein	1.96	1.70E−02
	Clocel_1430	Glycoside hydrolase family protein	Alpha-d-xyloside xylohydrolase	1.90	9.48E−03
	Clocel_2573	Hypothetical protein Clocel_2573	Chemotaxis protein CheX	1.60	3.95E−03
	Clocel_2592	Two component transcriptional regulator, AraC family	–	3.72	1.49E−04
	Clocel_2595	Xylan 1,4-beta-xylosidase	Xylan 1,4-beta-xylosidase	4.70	4.53E−06
	Clocel_2596	Sugar ABC transporter periplasmic protein	Ribose transport system substrate-binding protein	4.62	1.06E−03
	Clocel_2597	Inner-membrane translocator	Ribose transport system permease protein	4.01	7.36E−05
	Clocel_2598	ABC transporter	Ribose transport system ATP-binding protein	4.34	1.89E−05
	Clocel_2881	PTS system lactose/cellobiose-specific transporter subunit IIB	PTS system, cellobiose-specific IIB component	1.49	1.70E−02
	Clocel_2940	Putative phosphate transport regulator	Hypothetical protein	1.44	2.45E−02
	Clocel_3175	phoH family protein	phoH-like ATPase	1.64	3.84E−02
	Clocel_3761	ATP:guanido phosphotransferase	–	1.31	1.37E−02
	Clocel_3762	UvrB/UvrC protein	–	1.42	3.45E−02
	Clocel_4277	Aldo/keto reductase	–	1.08	4.66E−02
Galactomannan	Clocel_0034	Glycoside hydrolase family protein	Alpha-d-xyloside xylohydrolase	1.73	1.04E−02
	Clocel_0391	Glycosyltransferase	–	1.83	1.96E−02
	Clocel_0684	Thiazole biosynthesis protein ThiH	2-Iminoacetate synthase	1.57	4.69E−03
	Clocel_2225	Cobalamin (vitamin B12) biosynthesis CbiX protein	–	1.39	4.52E−02
	Clocel_2259	pfkB domain-containing protein	2-Dehydro-3-deoxygluconokinase	1.31	1.04E−02
	Clocel_2697	Sialate *O*-acetylesterase	Sialate *O*-acetylesterase	1.69	1.69E−02
	Clocel_2800	Alpha-galactosidase	Alpha-galactosidase	2.99	5.49E−05
	Clocel_2962	Inosine-5′-monophosphate dehydrogenase	IMP dehydrogenase	1.31	1.96E−02
	Clocel_3175	phoH family protein	phoH-like ATPase	1.98	1.14E−02
	Clocel_3194	Mannose-6-phosphate isomerase	Mannose-6-phosphate isomerase	3.77	6.86E−05
	Clocel_3196	Glycosidase-like protein	Beta-1,4-mannooligosaccharide/beta-1,4-mannosyl-*N*-acetylglucosamine phosphorylase	2.65	5.29E−03
	Clocel_3198	*N*-acylglucosamine 2-epimerase	Mannobiose 2-epimerase	3.19	2.10E−03
	Clocel_3200	Binding-protein-dependent transport system inner membrane protein	Multiple sugar transport system permease protein	4.76	8.61E−04
	Clocel_3201	Extracellular solute-binding protein	Raffinose/stachyose/melibiose transport system substrate-binding protein	4.23	5.49E−05
	Clocel_3205	Glycoside hydrolase family 2	Beta-mannosidase	2.55	1.51E−03
	Clocel_3657	Xylan 1,4-beta-xylosidase	Xylan 1,4-beta-xylosidase/alpha-*N*-arabinofuranosidase	1.79	4.26E−02
	Clocel_3857	ABC transporter	Multiple sugar transport system ATP-binding protein	2.15	1.03E−03
	Clocel_4053	LPXTG-motif cell wall anchor domain-containing protein	Sialate *O*-acetylesterase	2.28	3.79E−02
	Clocel_4087	Aldose 1-epimerase	Aldose 1-epimerase	1.45	2.68E−02
	Clocel_4088	Galactose-1-phosphate uridylyltransferase	UDP-glucose–hexose-1-phosphate uridylyltransferase	3.38	1.83E−04
	Clocel_4089	UDP-glucose 4-epimerase	UDP-glucose 4-epimerase	2.09	1.82E−03
	Clocel_4277	Aldo/keto reductase	–	1.08	4.26E−02
Pectin	Clocel_0048	Transcriptional regulator, AbrB family	Transcriptional pleiotropic regulator of transition state genes	1.91	2.50E−03
	Clocel_0322	tatD family hydrolase	tatD DNase family protein	1.74	8.66E−03
	Clocel_0513	Extracellular solute-binding protein	Raffinose/stachyose/melibiose transport system substrate-binding protein	2.12	2.50E−03
	Clocel_0519	Glycogen/starch/alpha-glucan phosphorylase	Starch phosphorylase	1.88	1.37E−02
	Clocel_1243	Extracellular solute-binding protein	Multiple sugar transport system substrate-binding protein	2.53	9.20E−05
	Clocel_1892	Acetate kinase	Acetate kinase	1.31	6.43E−03
	Clocel_2210	Nicotinate-nucleotide–dimethyl-benzimidazole phosphoribosyltransferase	Nicotinate-nucleotide–dimethyl-benzimidazole phosphoribosyltransferase	1.08	1.47E−02
	Clocel_2214	ATP:corrinoid adenosyltransferase BtuR/CobO/CobP	Coblalamin adenosyltransferase	1.37	4.25E−02
	Clocel_2222	Precorrin-3B C(17)-methyltransferase	Precorrin-3B C17-methyltransferase	1.70	6.63E−03
	Clocel_2225	Cobalamin (vitamin B12) biosynthesis CbiX protein	–	1.95	4.32E−03
	Clocel_2227	Precorrin-4 C(11)-methyltransferase	Precorrin-4/cobalt-precorrin-4 C11-methyltransferase	2.11	7.92E−03
	Clocel_2250	Altronate dehydratase	Altronate hydrolase	4.07	1.02E−05
	Clocel_2251	Mannitol dehydrogenase domain-containing protein	Tagaturonate reductase	4.50	4.39E−06
	Clocel_2253	Crp family transcriptional regulator	–	2.37	2.50E−03
	Clocel_2254	Glycoside hydrolase family protein	–	4.36	4.39E−06
	Clocel_2255	Major facilitator superfamily protein	Oligogalacturonide transporter	4.33	4.02E−05
	Clocel_2256	Glycosyl hydrolase family protein	Unsaturated rhamnogalacturonyl hydrolase	4.19	1.49E−05
	Clocel_2259	pfkB domain-containing protein	2-Dehydro-3-deoxygluconokinase	3.75	1.49E−06
	Clocel_2262	Short-chain dehydrogenase/reductase SDR	Gluconate 5-dehydrogenase	3.21	2.11E−02
	Clocel_2263	4-Deoxy-l-threo-5-hexosulose-uronate ketol-isomerase	4-Deoxy-l-threo-5-hexosulose-uronate ketol-isomerase	3.15	4.92E−03
	Clocel_2403	Glucosamine/fructose-6-phosphate aminotransferase	Glucosamine–fructose-6-phosphate aminotransferase (isomerizing)	1.45	4.57E−03
	Clocel_2737	Small GTP-binding protein	–	1.10	2.98E−02
	Clocel_3380	LPXTG-motif cell wall anchor domain-containing protein	–	4.03	1.27E−04
	Clocel_3909	Quorum-sensing autoinducer 2 (AI-2), LuxS	*S*-ribosylhomocysteine lyase	1.19	2.34E−02
	Clocel_4088	Galactose-1-phosphate uridylyltransferase	UDP-glucose–hexose-1-phosphate uridylyltransferase	1.62	1.73E−02
	Clocel_4277	Aldo/keto reductase	–	1.31	8.08E−03

To standardize data, obtained data were normalized with the median for all identified quantitative data. *P* values were adjusted with the Benjamini–Hochberg method to avoid the problem of multiple testing. For thresholds, we adopted an FDR-adjusted *p* value of <0.05 and fold change of protein ratio >2.0, compared to glucose.

### Profiles of metabolism-related proteins

First, we focused on profiles of metabolism-related proteins, such as enzymes involved in substrate degradation and metabolism, and other characteristic metabolic pathway. *C. cellulovorans* is known to change production of carbohydrate-related enzymes secreted into media from exoproteome analyses (Esaka et al. 2015; Matsui et al. 2013) and alternation of production of different metabolic pathways depending on which substrates are available in culture is also predicted.

### Degradation and metabolism of each substrate

**Figure 4:**
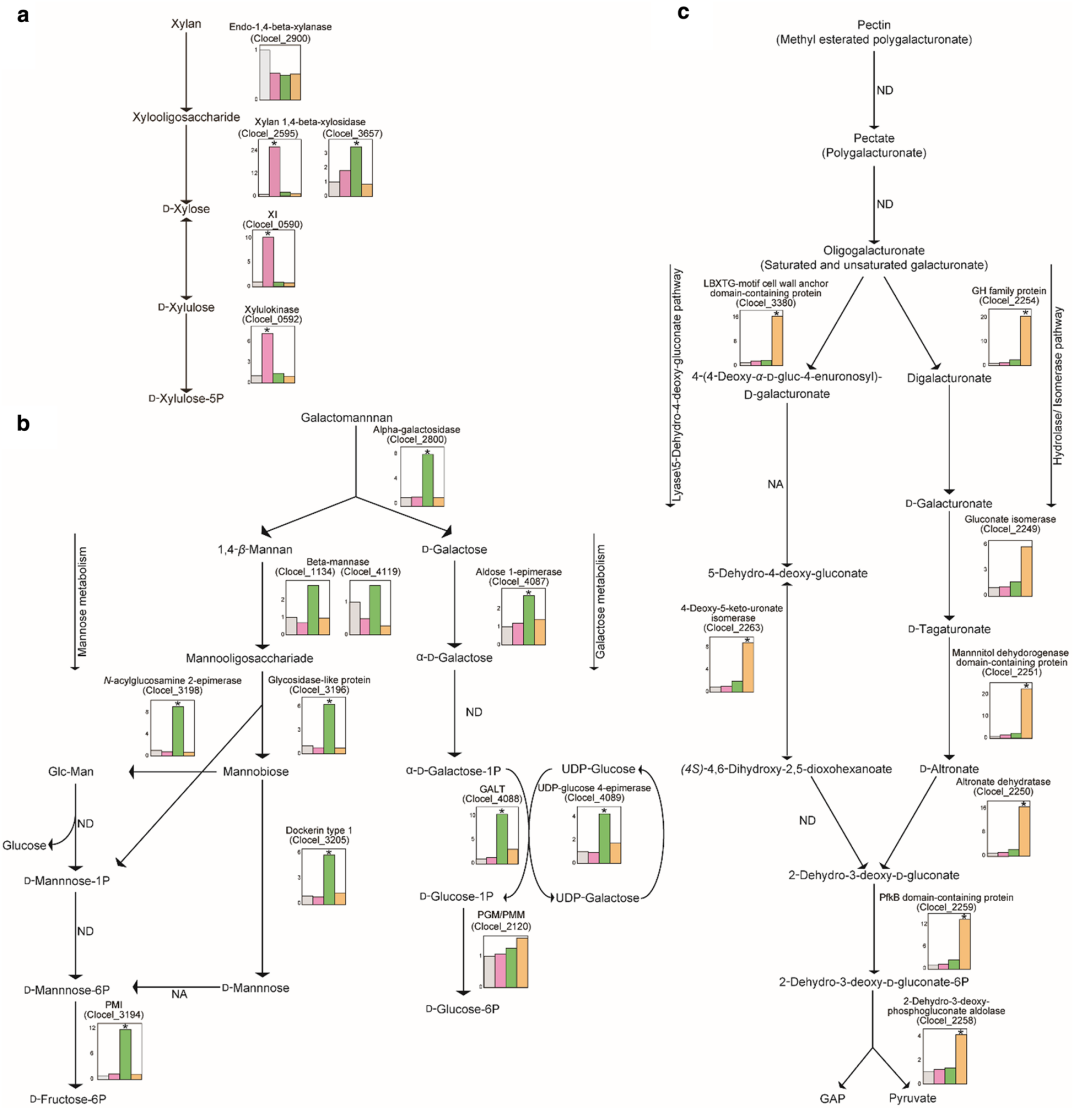
Degradation and metabolism pathways for each substrate, constructed from KEGG analysis. Each substrate degradation and metabolism pathway is shown: **a** xylan, **b** galactomannan (LBG), **c** pectin. For each protein, the fold change compared to glucose is shown. *Asterisk* denotes that protein levels are significantly elevated; as threshold, fold change >2.0, and FDR-adjusted *p* value <0.05 are adopted. *ND* protein not detected in this analysis. *NA* genes commonly assigned to the pathway, but not annotated. Fold change in glucose-grown cells is shown with a *gray bar* (=1); fold change in xylan-grown cells is shown with a *pink bar*; fold change of galactomannan-grown cells is shown with a *green bar*; fold change in pectin-grown cells is shown with an *orange bar*. *XI* xylose isomerase, *PMI* phosphomannose isomerase, *GALT* galactose-1-phosphate uridyltransferase, *PGM/PMM* phosphoglucomutase/phosphomannmutase alpha/beta/alpha domain I, *1P* 1-phosphate, *6P* 6-phosphate.

We constructed a substrate degradation pathway from KEGG pathway maps, and presented the fold change of each protein (Figure 4). For xylan degradation- and metabolism-related proteins (Figure 4a), the levels of three proteins (Clocel_0590, 0592, and 2595) were significantly elevated in the presence of xylan (Table 1), but production of Clocel_2900 (Endo-1, 4-beta xylanase) was not specifically elevated (Additional file 1: Table S1).

For galactomannan degradation- and metabolism-related proteins (Figure 4b), the levels of 9 proteins (Clocel_2259, 2800, 3194, 3196, 3198, 3205, 4087, 4088, and 4089) were significantly elevated in the presence of galactomannan (Table 1). By contrast, the levels of mannanase (Clocel_1134 and 4119) were not significantly elevated (Additional file 1: Table S1).

For pectin degradation- and metabolism-related proteins (Figure 4c), levels of 8 proteins (Clocel_2250, 2251, 2254, 2256, 2259, 2262, 2263, and 3380) were significantly elevated in the presence of pectin (Table 1). For the degradation and metabolism of pectin, both the “hydrolase/isomerase pathway” and the “lyase/5-dehydro-4-deoxy-gluconate pathway” were found (Richard and Hilditch 2009). Clocel_2254 is a member of the glycoside hydrolases (GH) 28 family (Cantarel et al. 2009), which is known to have endogalacturonase and exogalacturonase activities. Additionally, Clocel_3380 is a member of the polysaccharide lyase (PL) 9 family. BLAST analysis indicates that the protein has exogalacturonate lyase activity.

### Genome and cluster analyses

To identify candidates related to substrate recognition systems, we performed genomic analysis based on *C. cellulolyticum* TCS-related gene clusters (Xu et al. 2013). For the thresholds of TCS-regulated cluster identification, we applied two criteria. First, a cluster must contain more than two components of a TCS pathway: an integral membrane histidine kinase (sensor histidine kinase), a transcriptional regulator (response regulator), and an extracellular solute-binding protein (sugar binding protein). Second, degradation-, metabolism-, or transport-related gene loci must be located within 2 of the TCS genes identified above. Using this threshold, we identified 14 candidate clusters related to TCS.

**Figure 5:**
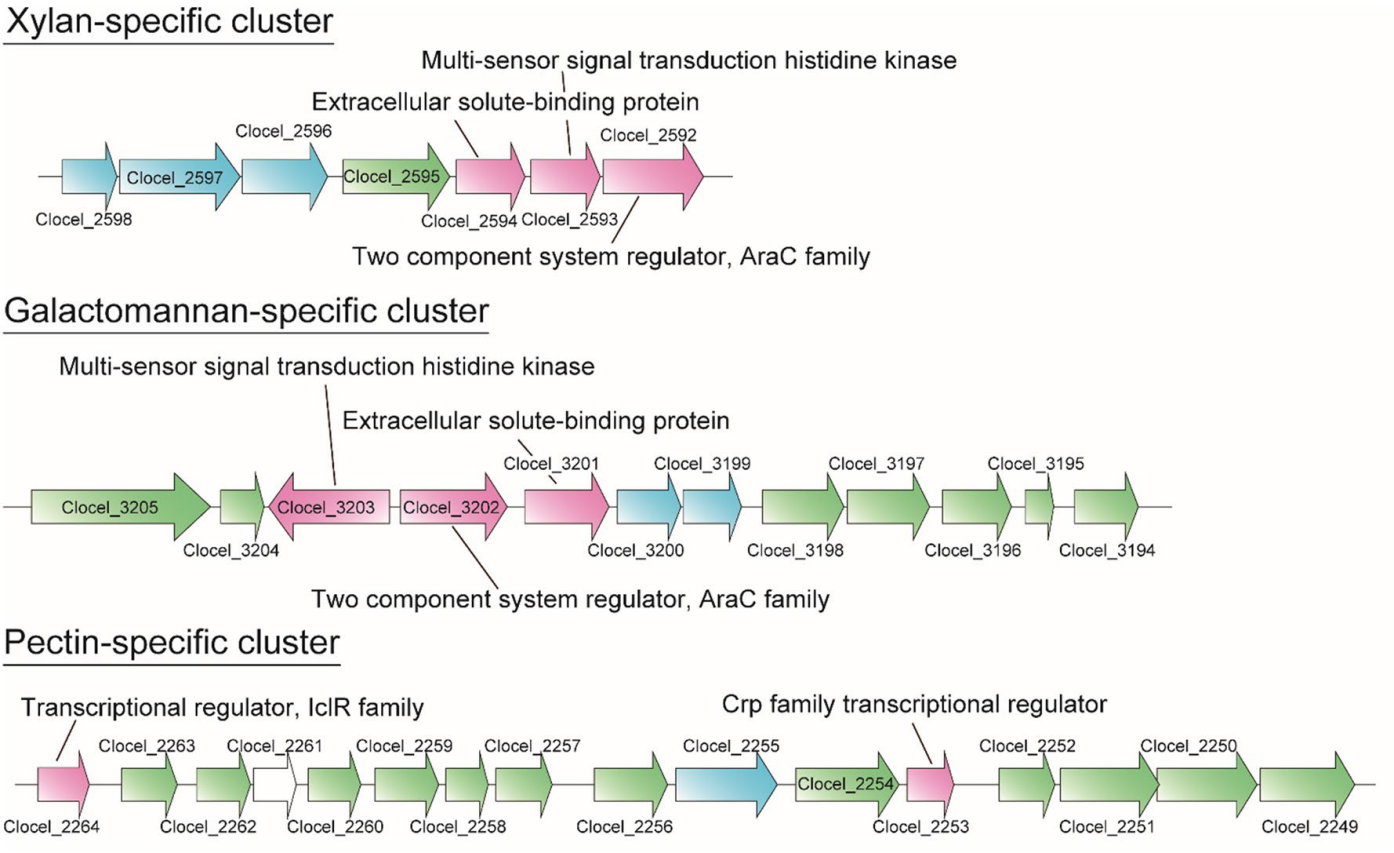
Candidates for gene clusters related to substrate recognition. We carried out genome analysis and identified candidates for gene clusters involved in TCS. Combining these results and substrate specific proteins, we found substrate-specific gene clusters related to recognition of each substrate. *Green arrows* indicate metabolism-related proteins, *pink arrows* indicate signal transduction-related proteins, and *blue arrows* indicate transport-related proteins. *White arrows* indicate pseudogenes.

Combining the information of our genome analysis and the substrate-specific proteins, we identified xylan- and galactomannan-specific clusters that include TCS-related genes. We identified clusters corresponding to two substrates; the xylan-specific cluster included Clocel_2592, 2595, 2596, 2597, and 2598 (Table 1), and Clocel_2593 and Clocel_2594 (Additional file 2: Table S2). The galactomannan-specific cluster included Clocel_3194, 3196, 3198, 3200, 3201, and 3205 (Table 1), and Clocel_3195, 3197, 3199, 3202, 3203, 3204 (Figure 5; Additional file 2: Table S2). Each cluster includes three components of a TCS: a transcriptional regulator AraC, an integral membrane histidine kinase, and an extracellular solute-binding protein. We suggest that these genes are common components of a hemicellulose recognition system.

From pectin, we found increased levels of proteins Clocel_2250, 2251, 2253, 2254, 2255, 2256, 2259, 2262, and 2263 (Table 1) and Clocel_2249, 2257, 2258, and 2260 (Additional file 2: Table S2). We suggest that this is a large cluster for the degradation and metabolism of pectin. For the degradation and metabolism of pectin, hydrolase/isomerase and lyase/5-dehydro-4-deoxy-gluconate pathways are known. The pectin-specific cluster which we identified contains genes related to both of these pathways, as well as for transporting pectin.

We also identified a xylose-metabolic cluster containing proteins Clocel_0589, 0590, 0591, and 0592 (Table 1) that had increased levels specifically in the presence of xylan. A galactose-metabolic cluster containing proteins Clocel_4087, 4088, and 4089 (Table 1) and Clocel_4090 (Additional file 2: Table S2) had elevated levels specifically in the presence of galactomannan. The xylose-metabolic cluster contained two xylose metabolism-related proteins, Clocel_0590 and 0592 (Table 1; Figure 4a), transaldorase (Clocel_0591; Table 1), which is related to the pentose phosphate pathway, and alpha-fucosidase (Clocel_0589; Table 1). In the galactose-metabolic cluster, galactose metabolism-related genes are present (Figure 4b) and increased levels of proteins Clocel_4087, 4088, and 4089 (Table 1) and Clocel_4090 (Additional file 2: Table S2) were found.

## Discussion

In total, we identified 734 proteins using our “intracellular” quantitative proteome analysis, and we were able to identify substrate-specific proteins using statistical analysis. Our method provided reproducibility, as evaluated by HCA (Figure 3). For substrate-specific proteins, we carried out genome analysis and cluster analysis following genome analysis on the basis of the results from our proteome analysis.

We first focused on metabolic pathways of each substrate in *C. cellulovorans* by KEGG analysis. For xylan metabolism, it is interesting to note that intracellular levels of Clocel_2595 (xylosidase) increased, but levels of Clocel_2900 (xylanase) were not enhanced. Although Clocel_2595 (xylosidase) and some xylanases were categorized as xylan-specific in “exoproteome” analysis (Matsui et al. 2013), *C. cellulovorans* cannot grow in medium containing xylose as the sole carbon source (Sleat et al. 1984). Combining our findings with the inability of *C. cellulovorans* to grow in xylose medium, we predict that *C. cellulovorans* metabolizes xylooligosaccharides intracellularly. We hypothesize the following pathway for xylan metabolism: *C. cellulovorans* degrades xylan to xylooligosaccharides extracellularly and transports them into the cytoplasm. Next, xylooligosaccharides are degraded to xylose in the cell, and xylose is metabolized and used by the pentose phosphate pathway. Indeed, we observed increased levels of Clocel_0591 (transaldolase), which is related to the pentose phosphate pathway, in xylan medium.

For galactomannnan metabolism, we focused on Clocel_3196 (glycosidase-like protein), which is a member of the GH130 family and is annotated as beta-1,4-mannooligosaccharide/beta-1,4-mannosyl-*N*-acetylglucosamine phosphorylase (Kawahara et al. 2012). Similarly annotated genes are part of mannan metabolic pathways in other bacteria (Senoura et al. 2011), and we observed increased levels of proteins in this pathway (Clocel_3198, 3205) in the presence of galactomannan. However, Clocel_3197, which is known to play an important role in the mannan metabolic pathway (Esaka et al. 2015), was only identified in our qualitative proteome analysis (Additional file 2: Table S2). This protein has been also detected with “exoproteome” analysis (Esaka et al. 2015). It is assumed that this protein acts both intracellularly and extracellularly through an unknown mechanism. In addition, we identified increased levels of alpha-galactosidase (Clocel_2800). In a previous study, this protein was identified in an “exoproteome” analysis, and it is predicted to degrade galactose side chains both intracellularly and extracellularly. Specifically, the extracellular collaboration between mannase and alpha galactosidase rapidly degrades galactomannan. Next, galactose side chains that were not degraded extracellularly are degraded by “intracellular” alpha galactosidase, and the resulting mannooligosaccharides and galactose are further metabolized.

We predicted a pectin metabolism pathway in *C. cellulovorans* by combining the results of the present study and the previously reported “exoproteome” analysis (Matsui et al. 2013). Extracellularly, pectin is degraded by pectin lyase (Clocel_0873, 1172, and 3380) to oligogalacturonate with both saturated and unsaturated ends. In parallel, pectin is also de-esterified to pectate (Clocel_0211 and 3114), resulting in simpler extracellular degradation pathways. The resulting metabolites are transported into the cell and metabolized with the hydrolase/isomerase and lyase/5-dehydro-4-deoxy-gluconate pathways. In *Erwinia chrysathemi*, which is a well-studied pathogenic bacterium, pectin is degraded using similar systems; i.e. pectin is degraded to oligogalacturonides outside of the cell. Next, oligogalacturonides are transported into cells and metabolized in the hydrolase/isomerase and lyase/5-dehydro-4-deoxy-gluconate pathways (Hugouvieux-Cotte-Pattat et al. 1996). While known for *E. chrysathemi*, this system has not been reported in other cellulosome-producing Clostridia. *C. cellulovorans* commonly degrades all sugar-substrates (polysaccharides) to oligosaccharides, not to monosaccharide, outside of the cell. Degradation of those oligosaccharides to monosaccharides and further metabolism take place intracellularly. Similar degradation systems have not been reported in Clostridia although *C. cellulolyticum* can metabolize xylose as a sole carbon source. Furthermore, in the presence of pectin, we found increased levels of an oligogalacturonides (esterified-oligogalacturonates) transporter (Clocel_2255). In the current analysis, we did not identify pectinesterase “intracellularly”, although it had previously been identified in an “exoproteome” analysis (Matsui et al. 2013). Together, these results suggest that oligogalacturonates are transported into *C. cellulovorans*, and degraded to monogalacturonates and further metabolized intracellularly. In addition to the degradation and metabolism of each substrate, levels of four proteins related to the synthesis of vitamin B12 were significantly elevated in cells cultured on pectin (Clocel_2214, 2222, 2225, and 2227). We are unable to find any pectin-specific proteins reported to have a requirement for vitamin B12 (Sañudo-Wilhelmy et al. 2014). For the degradation and metabolism of pectin, *C. cellulovorans* uses pectin lyase (Clocel_2259) (EC 4.2.1.7), which showed elevated levels in our analysis. We speculate that this enzyme may require vitamin B12.

Within the pectin clusters, a Crp family transcriptional regulator (Clocel_2253) was identified upstream of the hydrolase/isomerase pathway, and levels of this regulator were significantly increased. Crp family transcriptional regulators are known to regulate gluconate-related genes in *Escherichia coli* (Peekhaus and Conway 1998), and we propose that this protein similarly regulates the hydrolase/isomerase pathway in *C. cellulovorans*. Upstream of *Clocel_2263*, an IclR family transcriptional regulator encoded genes (*Clocel_2264*) was annotated (Figure 5). In our quantitative proteome analysis, we did not identify this protein, but in the qualitative proteome analysis, this protein was identified and displayed higher peptide spectrum matches (PSMs) in pectin than in xylan and galactomannan (Additional file 2: Table S2). The KdgR protein regulates the degradation of pectin in *Erwinia chrysanthemi*, and is in the IclR family transcriptional regulator group (Molina-Henares et al. 2006). BLAST analysis indicates that Clocel_2264 has similarity with the KdgR protein (e = 5E-48). Based on these findings, we proposed that IclR family transcriptional regulators control the expression of the lyase/5-dehydro-4-deoxy-gluconate pathway.

**Figure 6:**
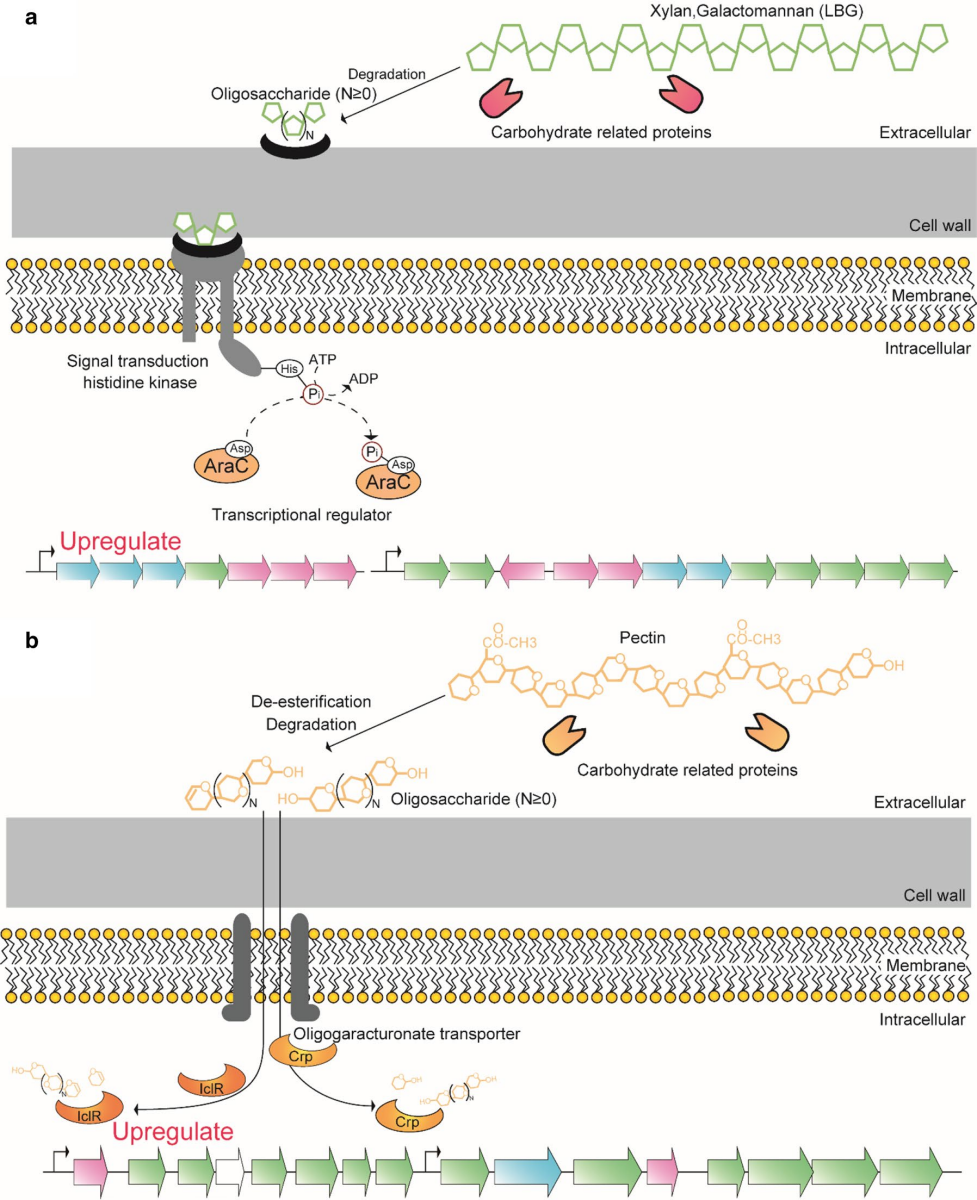
Proposed substrate recognition systems of hemicellulose and pectin in *C. cellulovorans*. For hemicellulose, polysaccharides are degraded to derived oligosaccharides outside of the cell, and extracellular solute-binding proteins bind these substrates. Solute-binding proteins induce signal-to-signal transduction integral membrane histidine kinases. Next the activated kinases phosphorylate transcriptional regulator AraC, and target genes (shown in Figure 5) are upregulated (**a**). For pectin, polysaccharides are de-esterified by pectinesterase and degraded by pectin lyase. Next, derived oligosaccharides are transported into cell, and these or other metabolites bind to a transcriptional regulator. Then, genes belonging to the target cluster (shown in Figure 5) are upregulated (**b**).

Based on the metabolism-related protein profiles and cluster analysis performed in the present study, we propose molecular models for substrate recognition of *C. cellulovorans* in hemicellulose (xylan and galactomannan) and pectin (Figure 6). For hemicellulose, polysaccharides (xylan and galactomannan) are first degraded to oligosaccharides (xylooligosaccharides and mannooligosaccharides) in the extracellular environment, and these oligosaccharides are recognized by extracellular solute binding proteins (Clocel_2594 in Additional file 2: Table S2; Clocel_3201 in Table 1). Oligosaccharide-binding proteins then transduce signals to integral membrane histidine kinases (Clocel_2593 and 3203 in Additional file 2: Table S2). Next, intracellular transcriptional regulators (Clocel_2592 in Table 1; Clocel_3202 in Additional file 2: Table S2) are phosphorylated by the activation of integral membrane histidine kinases. Finally, genes related to the degradation and metabolism of hemicellulose are upregulated, allowing *C. cellulovorans* to metabolize substrates more efficiently (Figure 6a).

For pectin, two regulators (Clocel_2253 in Table 1; Clocel_2264 in Additional file 2: Table S2) work in substrate recognition. We predict that oligosaccharides derived from pectin or other pectin metabolites bind to these regulators allowing oligosaccharides to be transported into the cell (Figure 6b). This is a similar system to the pectin-recognition system in *E*. *chrysathemi* (Molina-Henares et al. 2006), but *E*. *chrysathemi* does not have two regulators operating hydrolase/isomerase and lyase/5-dehydro-4-deoxy-gluconate pathways.

The xylan recognition system of *C. cellulovorans* is very similar to that of *C. cellulolyticum*, but based on previous research (Xu et al. 2013), the composition of clusters is clearly different. In the *C. cellulolyticum* cluster, metabolism-related enzymes were not included. We therefore predict that these genes may not be regulated by TCS (Xu et al. 2013). However, the *C. cellulovorans* cluster includes metabolism- and transport-related genes. Thus, we speculate that the recognition system for xylan in *C. cellulovorans* regulates both the production of degradation- and transport-related enzymes and metabolism-related enzymes.

Substrate recognition systems in *C. cellulovorans* are one of the survival strategies. In other bacteria, monosaccharides such as glucose and xylose are mainly transported, but this bacterium recognizes oligosaccharides of hemicellulose and pectin, and can optimize degradation-, metabolism-, and transport-related proteins’ profiles according to the substrate faster than other bacteria. This feature maybe suitable for competing with other anaerobic bacteria.

In conclusion, we performed “intracellular” quantitative proteome analysis of *C. cellulovorans* with glucose, xylan, galactomannan, and pectin and identified 734 proteins with LC–MS/MS equipped long monolithic silica capillary columns. We focused on substrate-recognition-, degradation-, and metabolism-related proteins, and carried out cluster analysis based on genome analysis together with the intracellular proteome analysis and the previous “exoproteome” analysis (Matsui et al. 2013; Esaka et al. 2015). Based on our results, we propose a mechanism for the recognition of hemicellulose and pectin in *C. cellulovorans*. For hemicellulose recognition, *C. cellulovorans* uses TCS and regulates all substrate-related genes with a single regulator. In the pectin recognition system, *C. cellulovorans* regulates hydrolase/isomerase and lyase/5-dehydro-4-deoxy-gluconate pathways with two regulators, Clocel_2253 and Clocel_2264, respectively. Judging from the presence of an oligogalacturonate transporter in the pectin regulatory clusters, the regulation of pectin metabolism-related genes appears to be controlled by the oligogalacturonates derived from pectin, or other metabolic products. This system has not been described for other cellulosome-producing Clostridia. Due to the multiplicity of regulator proteins involving substrate recognition, we suggest that *C. cellulovorans* has more distinct promoter regions that respond to each substrate. This information should be helpful for the elucidation of the environmental sensing strategy of *C. cellulovorans*.

## Additional files

Additional file 1: Table S1. The 734 proteins identified in quantitative proteome analysis. Proteome analytes were injected to LC–MS/MS system. Obtained data were used for protein identification with Proteome Discoverer software 1.4. Three independent biological experiments were carried out, and proteins with no missing values were accepted as identified protein. As a result, 734 proteins were successfully identified. Additional file 2: Table S2. All proteins identified in the qualitative proteome analysis. Supplemental qualitative proteome analysis was carried out to strengthen the quantitative proteome analysis. Three independent biological experiments were carried out, and proteins with MASCOT scores >10 were accepted as identified protein. Score: MASCOT score; PSMs: Peptides spectrum matches.

## Abbreviations

ATCC: American type culture collection
CID: collision induced dissociation
EC: enzyme commission number
ESI: electrospray ionization
FDR: false discovery rate
GH: glycoside hydrolases
HCA: hierarchical cluster analysis
HCD: higher-energy c-trap dissociation
HPLC: high performance liquid chromatography
ID: internal diameter
KEGG: Kyoto encyclopedia of genes and genomes
LBG: locust bean gum
LC–MS: liquid chromatography–mass spectrometry
NCBI: National Center for Biotechnology Information
PBS: phosphate buffered saline
PL: polysaccharide lyase
SDS: sodium dodecyl sulfate
TCS: two component system
TEAB: triethyl ammonium bicarbonate
TMT: tandem mass tag

## References

[CR1] Aoki W, Ueda T, Tatsukami Y, Kitahara N, Morisaka H, Kuroda K (2013). Time-course proteomic profile of *Candida albicans* during adaptation to a fetal serum. Pathog Dis.

[CR2] Cantarel BL, Coutinho PM, Rancurel C, Bernard T, Lombard V, Henrissat B (2009). The carbohydrate-active enzymes database (CAZy): an expert resource for glycogenomics. Nucleic Acids Res.

[CR3] Celik H, Blouzard J-C, Voigt B, Becher D, Trotter V, Fierobe H-P (2013). A two-component system (XydS/R) controls the expression of genes encoding CBM6-containing proteins in response to straw in *Clostridium cellulolyticum*. PLoS One.

[CR4] Collins M, Lawson P, Willems A, Cordoba J, Fernandez-Garayzabal J, Garcia P (1994). The phylogeny of the genus Clostridium: proposal of five new genera and eleven new species combinations. Int J Syst Bacteriol.

[CR5] Cosgrove DJ (2005). Growth of the plant cell wall. Nat Rev Mol Cell Biol.

[CR6] de Hoon MJ, Imoto S, Nolan J, Miyano S (2004). Open source clustering software. Bioinformatics.

[CR7] Esaka K, Aburaya S, Morisaka H, Kuroda K, Ueda M (2015). Exoproteome analysis of *Clostridium cellulovorans* in natural soft-biomass degradation. AMB Express.

[CR8] Higashide W, Li Y, Yang Y, Liao JC (2011). Metabolic engineering of *Clostridium cellulolyticum* for production of isobutanol from cellulose. Appl Environ Microbiol.

[CR9] Hugouvieux-Cotte-Pattat N, Condemine G, Nasser W, Reverchon S (1996). Regulation of pectinolysis in *Erwinia chrysanthemi*. Annu Rev Microbiol.

[CR10] Kanehisa M, Goto S (2000). KEGG: kyoto encyclopedia of genes and genomes. Nucleic Acids Res.

[CR11] Kawahara R, Saburi W, Odaka R, Taguchi H, Ito S, Mori H (2012). Metabolic mechanism of mannan in a ruminal bacterium, *Ruminococcus albus*, involving two mannoside phosphorylases and cellobiose 2-epimerase: discovery of a new carbohydrate phosphorylase, β-1,4-mannooligosaccharide phosphorylase. J Biol Chem.

[CR12] Lynd LR, Wyman CE, Gerngross TU (1999). Biocommodity engineering. Biotechnol Prog.

[CR13] Matsui K, Bae J, Esaka K, Morisaka H, Kuroda K, Ueda M (2013). Exoproteome profiles of *Clostridium cellulovorans* grown on various carbon sources. Appl Environ Microbiol.

[CR14] Molina-Henares AJ, Krell T, Guazzaroni ME, Segura A, Ramos JL (2006). Members of the IclR family of bacterial transcriptional regulators function as activators and/or repressors. FEMS Microbiol Rev.

[CR15] Morisaka H, Matsui K, Tatsukami Y, Kuroda K, Miyake H, Tamaru Y (2012). Profile of native cellulosomal proteins of *Clostridium cellulovorans* adapted to various carbon sources. AMB Express.

[CR16] Nataf Y, Bahari L, Kahel-Raifer H, Borovok I, Lamed R, Bayer EA (2010). *Clostridium thermocellum* cellulosomal genes are regulated by extracytoplasmic polysaccharides via alternative sigma factors. Proc Natl Acad Sci USA.

[CR17] Peekhaus N, Conway T (1998). Positive and negative transcriptional regulation of the *Escherichia coli* gluconate regulon gene gntT by GntR and the cyclic AMP (cAMP)-cAMP receptor protein complex. J Bacteriol.

[CR18] Petitdemange E, Caillet F, Giallo J, Gaudin C (1984). *Clostridium cellulolyticum* sp. nov., a cellulolytic, mesophilic: species from decayed grass. Int J Syst Bacteriol.

[CR19] Prawitwong P, Waeonukul R, Tachaapaikoon C, Pason P, Ratanakhanokchai K, Deng L (2013). Direct glucose production from lignocellulose using *Clostridium thermocellum* cultures supplemented with a thermostable β-glucosidase. Biotechnol Biofuels.

[CR20] Rainey FA, Stackebrandt E (1993). 16S rDNA analysis reveals phylogenetic diversity among the polysaccharolytic clostridia. FEMS Microbiol Lett.

[CR21] Raman B, Pan C, Hurst GB, Rodriguez M, McKeown CK, Lankford PK (2009). Impact of pretreated switchgrass and biomass carbohydrates on *Clostridium thermocellum* ATCC 27405 cellulosome composition: a quantitative proteomic analysis. PLoS One.

[CR22] Richard P, Hilditch S (2009). d-Galacturonic acid catabolism in microorganisms and its biotechnological relevance. Appl Microbiol Biotechnol.

[CR23] Saha BC (2003). Hemicellulose bioconversion. J Ind Microbiol Biotechnol.

[CR24] Saldanha AJ (2004). Java Treeview—extensible visualization of microarray data. Bioinformatics.

[CR25] Sañudo-Wilhelmy SA, Gómez-Consarnau L, Suffridge C, Webb EA (2014). The role of B vitamins in marine biogeochemistry. Ann Rev Mar Sci.

[CR26] Senoura T, Ito S, Taguchi H, Higa M, Hamada S, Matsui H (2011). New microbial mannan catabolic pathway that involves a novel mannosylglucose phosphorylase. Biochem Biophys Res Commun.

[CR27] Sleat R, Mah RA, Robinson R (1984). Isolation and characterization of an anaerobic, cellulolytic bacterium, *Clostridium cellulovorans* sp. nov. Appl Environ Microbiol.

[CR28] Smyth GK (2004) Linear models and empirical bayes methods for assessing differential expression in microarray experiments. Stat Appl Genet Mol Biol 3(1) 10.2202/1544-6115.1027 16646809

[CR29] Tamaru Y, Miyake H, Kuroda K, Nakanishi A, Kawade Y, Yamamoto K (2010). Genome sequence of the cellulosome-producing mesophilic organism *Clostridium cellulovorans* 743B. J Bacteriol.

[CR30] Xu C, Huang R, Teng L, Wang D, Hemme CL, Borovok I (2013). Structure and regulation of the cellulose degradome in *Clostridium cellulolyticum*. Biotechnol Biofuels.

